# Advances in Molecular Mechanisms Underlying Cadmium Accumulation and Detoxification in Durum Wheat (*Triticum turgidum* L. subsp. *durum* (Desf.) Husn.)

**DOI:** 10.3390/ijms27135802

**Published:** 2026-06-26

**Authors:** Sami ur Rehman, Michele Benedetti, Ignazio Allegretta, Alessio Aprile

**Affiliations:** 1 Department of Biological and Environmental Sciences and Technologies, University of Salento, Via Monteroni 165, 73100 Lecce, Italy; sami.urrehman@unisalento.it (S.u.R.); ignazio.allegretta@unisalento.it (I.A.); 2 Department of Engineering for Innovation, University of Salento, Via Monteroni 165, 73100 Lecce, Italy

**Keywords:** metal transporters, cadmium uptake, durum wheat, cadmium detoxification, molecular mechanism

## Abstract

Cadmium (Cd) pollution adversely affects crop productivity and grain quality. Durum wheat (*Triticum turgidum* L. subsp. *durum* (Desf.) Husn.), a widely consumed cereal crop, can accumulate substantial levels of Cd in edible tissues, threatening human health. Therefore, advances in understanding Cd toxicity in plants and the molecular mechanisms underlying Cd accumulation and detoxification are needed to develop resistant cultivars and ensure safe food production. Although Cd homeostasis has been previously studied in bread wheat, its accumulation varies among species and cultivars owing to differences in their physiological and genetic makeup. However, the molecular mechanisms underlying Cd homeostasis in durum wheat have not been comprehensively reviewed. Here, we synthesize current knowledge on the molecular basis of Cd uptake, transport, and detoxification in durum wheat. Specialized transporter families, including MRPs/ABCCs, HMAs, and members of the YSL, ZIFL, and IREG families, play critical roles in mediating Cd compartmentalization and limiting its cytosolic toxicity. Genes such as *HMT1*, *TdHMA3-B1a*, and members of the NAS gene family significantly reduced Cd accumulation in grains. Future studies should focus on the integration of physiological, molecular genetics, and multi-omics approaches to uncover the regulatory networks controlling Cd homeostasis in durum wheat.

## 1. Introduction

Soil contamination with heavy metals, particularly Cd, poses a serious threat to crop productivity and human health. Cadmium is widely distributed in the Earth’s crust and is released into the environment through both natural processes and human activities, including industrial operations and agricultural practices [1]. Agricultural soils are increasingly contaminated with Cd, mainly because of atmospheric deposition from mining activities, particularly those involving lead (Pb), zinc (Zn), and copper (Cu), as well as the extensive use of phosphate-based fertilizers [2,3].

Cadmium not being necessary for plant physiological function and its high mobility within the soil–plant system make it one of the most phytotoxic elements [4,5]. Among plant species, cereals are particularly prone to Cd accumulation in their grains, with more than 40% of the absorbed Cd being translocated to aerial tissues [6]. Durum wheat (*T. durum*) is an important tetraploid cereal crop with global annual production of approximately 33–40 million tonnes, accounting for about 6–7% of total wheat production worldwide. It is predominantly cultivated across the Mediterranean Basin and North America, with additional cultivation in Mexico, Russia, Kazakhstan, India, and Australia [7,8]. Due to its broad consumption, especially pasta and couscous, durum wheat represents a major dietary source of Cd [9]. To lessen health risks associated with Cd exposure, the European Union revised the maximum permissible levels of Cd in foodstuffs through Commission Regulation (EU) 2021/1323, amending Regulation (EC) No 1881/2006, and established a maximum admissible Cd concentration of 0.18 mg kg^−1^ fresh weight for durum wheat [10].

Cadmium stress substantially affected the physiological and molecular processes, leading to reduced growth and yield of durum wheat. Elevated Cd concentrations in soil induce oxidative stress in plants by promoting the overproduction of reactive oxygen species (ROS), including hydrogen peroxide (H_2_O_2_), superoxide radicals (O_2_^•−^), and hydroxyl radicals (•OH), which may severely harm cellular membranes, lipids, proteins, RNA, and DNA [5]. Furthermore, Cd uptake primarily overlaps with transport pathways for Fe, Zn, Mn, and Ca; thus, Cd exposure can indirectly affect the homeostasis of other essential nutrients, including magnesium (Mg) and Zinc (Zn), through competitive interactions and physiological imbalances [11,12].

Plants respond to Cd toxicity through multiple adaptive mechanisms, including (i) chelation, (ii) intracellular compartmentalization, and (iii) interactions with essential nutrients. The plant behavior to Cd exposure is highly complex and involves the coordinated action of transcription factors, metal chelators, heavy-metal transporters, antioxidant enzymes, and aquaporins, as demonstrated by numerous physiological and transcriptomic studies, including recent work in durum wheat [13]. Understanding the molecular mechanisms underlying Cd uptake, translocation, and detoxification in durum wheat is therefore essential for developing resistant cultivars with reduced Cd uptake.

Previous studies primarily focused on the mechanisms governing Cd uptake and translocation in common wheat and rice cultivars. However, mechanisms of Cd homeostasis vary significantly with plant species and even among wheat cultivars due to variations in their genetic makeup, root morphology, and physiological responses [14]. The durum wheat cultivar is of key concern, as it generally accumulates higher grain Cd levels as compared to bread wheat, posing serious threats to food safety [15]. Various studies elaborated on the recent advances in physiological, molecular and genetic networks in durum wheat [13,16,17]. However, these findings remain fragmented and are not integrated into a comprehensive review. To our knowledge, this is the first review that combines current knowledge on Cd uptake and homeostasis at physiological, molecular and genetic levels, critical to unravelling the regulatory networks responsible for minimizing Cd levels in durum wheat. Therefore, integration of these approaches is essential in developing efficient and low-Cd cultivars for sustainable durum wheat production in contaminated soils while meeting food safety standards.

## 2. Sources of Cadmium in Agricultural Land

The contamination of agricultural soil by heavy metals is one of the most pressing issues at present. According to recent meta-analyses, the major sources contributing to Cd accumulation in agricultural soils include atmospheric deposition (16–99%), application of mineral fertilizers (0–74%), and irrigation with contaminated water (0–76%), highlighting the substantial variability in Cd inputs across regions and agricultural systems [18]. Cadmium contamination in soils originates from a combination of lithogenic processes and anthropogenic activities. The main contribution of Cd from the Earth’s crust is due to natural weathering of rocks. Mafic and ultramafic rocks hold higher quantities of Cd, contributing substantially to Cd release into the soil upon weathering [19]. On a global scale, the environment receives approximately 25,000 tons of Cd annually through natural processes, including geological activities, volcanic emissions, and rock weathering [20]. Moreover, forest fires, volcanic eruptions, sea sprays, and wind-blown dust are natural contributors to Cd transfer to the environment [21].

**Figure 1:**
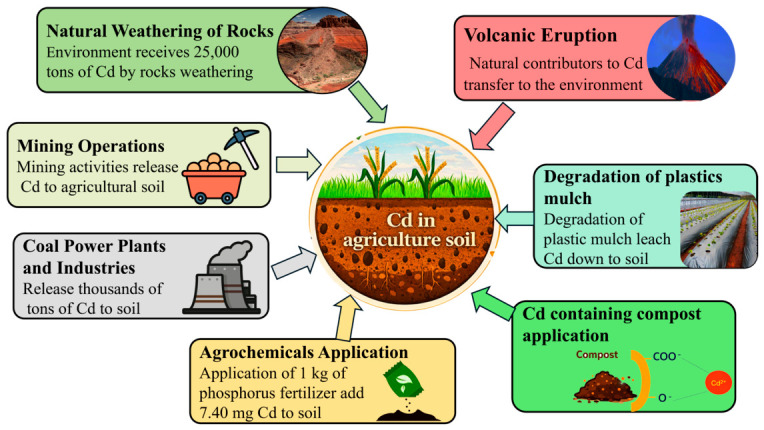
Major pathways of cadmium contamination in agricultural soils.

Anthropogenic inputs are a major source of Cd contamination in agricultural soils and primarily occur through atmospheric deposition, irrigation with contaminated wastewater, extensive use of agrochemicals, fertilizers, and amendments, and improper disposal of industrial and municipal waste (Figure 1). Additionally, Cd inputs arise from industrial waste associated with electroplating, plastics, pigment formulation, and nickel–cadmium battery disposal, as well as emissions from coal power plants [20,21,22,23,24].

Furthermore, leaching from biodegradable plastic films may represent a minor pathway contributing to Cd accumulation in agricultural soil [25]. Metals deposited on the soil surface can infiltrate the topsoil, increasing soil metal concentrations, whereas those deposited on plant surfaces may either remain adhered to foliar tissues or be washed into the soil by precipitation [26]. As a result of these combined anthropogenic activities, Cd concentrations in arable soils typically range from 0.06 to 1.10 mg kg^−1^, with an average concentration of approximately 0.41 mg kg^−1^ [27]. Amendments and agrochemicals are widely applied in modern agriculture to enhance soil fertility and crop productivity by supplying essential nutrients and protecting plants from pests and diseases. Long-term use of synthetic fertilizers, particularly phosphate-based fertilizers, can lead to contamination of agricultural soils with toxic trace metals such as Cd and Pb [28]. Additionally, a 50-year long-term field study by Park et al. [29] reported elevated Cd concentrations in the soils receiving repeated applications of compost and phosphorus fertilizers. Cadmium contamination in phosphate fertilizers primarily originates from the use of rock phosphate as a raw material, with Cd content ranging from 0.10 to 60 mg kg^−1^, depending on geological origin [30]. In Europe, the application of phosphate fertilizers represents a significant source of Cd inputs to agricultural soils, with reported average concentrations of approximately 7.40 mg Cd per kg of phosphate fertilizer [27].

In addition to mineral fertilizers, organic amendments such as manure and sewage sludge constitute supplementary sources of Cd input to agricultural land. Due to their relatively low concentrations of essential plant nutrients compared with synthetic fertilizers, these materials are often applied at higher rates to meet crop nutritional requirements, which may inadvertently enhance Cd accumulation in soils. Though Cd is persistent in the environment and is difficult to remediate once released. Cadmium leaching can contaminate aquatic environments, including surface waters used for irrigation, thereby facilitating Cd transfer to agricultural soils and its subsequent accumulation in crops [31]. At the global scale, soil Cd accumulation has been estimated to range between 5 and 38 × 10^6^ kg year^−1^, with agricultural and livestock-related wastes accounting for approximately 12% of the total input [21]. Furthermore, traces of Cd and other toxic metals may be present in livestock feed because of contamination of mineral supplements. Following ingestion, these metals are largely excreted via dung and urine, leading to their accumulation in manure, which is subsequently applied to agricultural land as an organic amendment. Reported Cd inputs to agricultural soils from manure application range from 1.4 to 6.1 g Cd ha^−1^ year^−1^ [26].

To comprehend the physiological mechanisms governing Cd uptake, translocation and redistribution in durum wheat grains, it is crucial to trace the pathways of metal movement into the soil and later to the reproductive parts of the plant [32]. Furthermore, Cd accumulation in durum wheat grains is genetically controlled and depends on Cd bioavailability in the rhizosphere, highlighting the importance of managing rhizosphere contamination to reduce Cd uptake in grain.

## 3. Cd Toxicity to Durum Wheat Growth

### 3.1. Effects of Cd on Seed Germination and Early Seedling Development

Cadmium toxicity in plants manifests early during seed germination and seedling establishment, affecting multiple physiological and biochemical processes. Previous studies have reported that Cd exposure adversely influences germination rate, seedling vigor, and early growth parameters in durum wheat [33]. Higher soil Cd concentrations (>5–10 mg kg^−1^), applied under controlled experimental conditions, markedly suppress seed germination and seedling growth [34,35].

The inhibitory effects of Cd on seed germination are primarily associated with impaired water uptake during imbibition, which restricts embryo expansion and delays radicle protrusion [36]. In addition, Cd presence in the rhizosphere alters root hair development and disrupts starch mobilization from the endosperm. This results in reduced hydrolysis of carbohydrate reserves and limited translocation of soluble sugars to the embryonic axis, ultimately impairing germination and early seedling growth [37].

During imbibition, increased permeability of the seed coat may further facilitate Cd entry into seed tissues, exacerbating its toxic effects and contributing to delayed or inhibited germination [38]. Collectively, these findings indicate that Cd interferes with key metabolic and structural processes required for successful seed germination and early seedling establishment in durum wheat.

### 3.2. Root System Impairment

The root system is the primary site of Cd contact and toxicity in plants, as roots are directly exposed to Cd present in the rhizosphere. Exposure to Cd rapidly disrupts root morphology both at macroscopic (increased number of tips and primary root length) and ultrastructural levels (cell membrane system damaged, cell walls thickened and enriched in suberin) [39]. These effects are mainly attributed to the direct interaction between Cd and root tissues, which threatens membrane integrity and interferes with the activity of water and nutrient transport systems.

Cadmium stress has also been associated with alterations in plant hormonal balance, particularly a reduction in cytokinin levels. Decreased cytokinin availability contributes to reduced transpiration and shoot growth, further worsening the negative effects of Cd on plant development [40]. Moreover, Cd-induced oxidative stress in root tissues affects the dynamics of cell division and differentiation within the root apical meristem. Elevated levels of reactive oxygen species (ROS) have been detected in the root apex of Cd-treated wheat seedlings, where they promote a premature transition of cells from the division zone to the elongation and differentiation zones, resulting in a shortened meristem and reduced root elongation [41,42]. Cd exposure markedly increased cell wall thickness, particularly in the pericycle and xylem. In Cd-treated roots, cell wall thickness reached ~3.5 µm, compared with ~1.6 µm in control plants [39]. The damage to the cell wall and disintegration of organelles were observed in roots exposed to Cd. Furthermore, Cd contamination resulted in damage and deformation of mitochondrial and nuclear membranes in wheat root tips [43].

Collectively, these physiological, anatomical, and developmental variations affect root system architecture and functions, thereby limiting the plant’s capability to acquire water and key nutrients and ultimately constrain overall growth under Cd stress.

### 3.3. Photosynthetic Dysfunction and Chloroplast Damage

**Figure 2:**
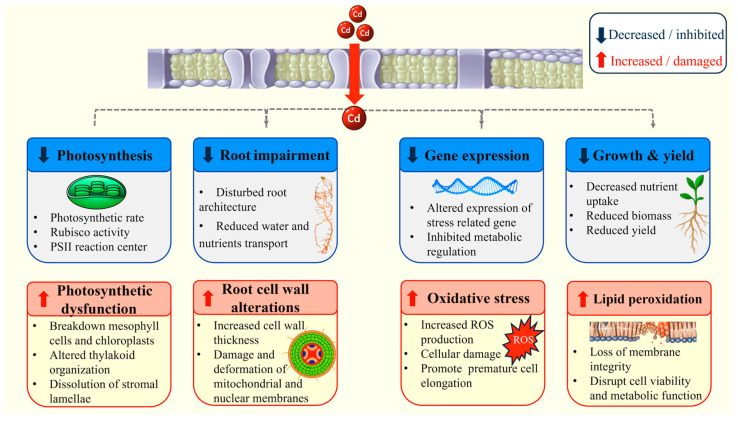
Overview of Cd uptake and its adverse effects on plant systems. Cd entry disrupts photosynthesis, root function, gene expression, and growth while inducing oxidative stress, cellular damage, and lipid peroxidation. These combined effects ultimately reduce plant productivity and viability under Cd stress.

Cadmium exposure severely impairs photosynthetic performance in durum wheat by changing both the physical integrity and functional efficiency of the photosynthetic apparatus (Figure 2). A reduction in plant growth under Cd stress is commonly associated with decreased chlorophyll content and inhibition of photosynthetic activity [44,45]. At elevated Cd concentrations, damage to the photosystem II (PSII) reaction center greatly reduces the net photosynthetic rate [46].

Cadmium interferes with the water-oxidizing complex of PSII by displacing Mn^2+^ ions, thereby limiting electron donation and impairing photochemical reactions [44]. In addition, Cd alters the activity of key enzymes involved in carbon fixation, including ribulose-1,5-bisphosphate carboxylase/oxygenase (RuBisCO) and phosphoenolpyruvate carboxylase. The substitution of Mg^2+^, a critical cofactor for RuBisCO activity, by Cd^2+^ disrupts enzyme structure and function, further constraining CO_2_ assimilation [47,48]. Cd-induced photosynthetic inhibition has been measured at the level of individual stomata, where Cd uptake was shown to reduce the photosynthetic rate by up to 26% [49]. Ultrastructural analyses have revealed that Cd stress promotes the disintegration of mesophyll cells and chloroplasts, characterized by reduced starch accumulation, altered thylakoid organization, and dissolution of stromal lamellae [50]. These structural variations are accompanied by a marked decline in the electron transport rate of PSII, the effective quantum yield of photochemical energy conversion, and photosynthetically active radiation utilization, ultimately abolishing photosynthetic efficiency [51]. Beyond chloroplast dysfunction, Cd also negatively affects mitochondrial and chloroplastic electron conduction chains, additionally worsening energy imbalance and metabolic disruption in plant cells [52].

### 3.4. Oxidative Stress and Cellular Damage

Although Cd is not a redox-active metal, its accumulation in plant tissues induces oxidative stress by disrupting cellular redox homeostasis. Exposure to Cd leads to the generation of ROS, such as H_2_O_2_, O_2_^•−^, and •OH, which surpass the antioxidant capacity of plant cells [41].

The overproduction of ROS causes extensive cellular damage, including lipid peroxidation of biological membranes, protein oxidation, and nucleic acid impairment, ultimately disrupting cell viability and metabolic function. In durum wheat, Cd-induced oxidative stress correlates with increased protein carbonylation and membrane lipid peroxidation, as well as a reduction in the ascorbate redox state [53,54]. These redox imbalances disrupt membrane integrity and enzyme activities, thereby amplifying Cd toxicity to plant growth and development.

Overall, oxidative stress constitutes a central mechanism by which Cd exerts cytotoxic effects, connecting primary metal exposure to subsequent physiological, metabolic, and developmental impairments in durum wheat.

### 3.5. Impact of Cd on Agronomic Traits and Yield

Soil Cd contamination exerts pronounced negative effects on key agronomic traits of wheat, ultimately leading to yield losses. Studies conducted on wheat genotypes with contrasting Cd tolerance have shown that Cd stress significantly reduces plant growth and enhances lipid peroxidation in leaf tissues [55].

In a comprehensive evaluation of 51 wheat varieties exposed to Cd-contaminated soil (20 mg kg^−1^), Rebekić and Lončarić [56] reported a general reduction in agronomic performance across all genotypes. The most severe declines were observed in grain and spike weight (up to 27%), followed by reductions in total plant biomass (25%) and number of grains per spike (23%). These findings highlight the sensitivity of yield-related traits to Cd stress and underscore the agronomic relevance of Cd toxicity in wheat production systems.

Cadmium accumulation also interferes with the uptake and metabolism of essential mineral nutrients, including Fe, Ca, Cu, and Zn, thereby exacerbating its negative effects on plant growth and productivity [57]. Antagonistic interactions between Cd and Zn have been associated with reductions in dry matter accumulation and grain yield in durum wheat. Elevated Cd levels combined with reduced Zn availability significantly impair biomass production and yield in wheat varieties [58].

Notably, some studies have reported limited or non-significant effects of Cd stress on wheat biomass and yield parameters [35,59]. Such variability may be attributed to genotype-dependent differences in Cd uptake and translocation, with Cd preferentially retained in root tissues and restricted movement to aerial organs in more tolerant genotypes. This highlights the importance of genetic factors governing Cd partitioning and tolerance in determining agronomic outcomes under Cd-contaminated conditions.

## 4. Cadmium Accumulation and Translocation in Durum Wheat

### 4.1. Root Cd Uptake: Physicochemical and Anatomical Determinants

Cadmium accumulation in durum wheat roots depends on soil properties, rhizosphere processes, and root anatomical traits. Cadmium bioavailability in soil is also influenced by pH, redox potential, soil organic matter, and nutrient composition, which regulate Cd solubility and mobility [60]. Cadmium in soil is distributed among different geological fractions, including soluble and exchangeable, complexed with Fe/Mn oxides and organic matter, and precipitated with carbonates [61]. The soluble and exchangeable fractions are considered bioavailable for plant uptake. In addition, the non-residual fractions, carbonates, Fe-Mn oxides, and organic matter fractions may also become bioavailable under changing soil conditions, particularly with variations in pH and redox potential [62]. Furthermore, low soil pH facilitates the desorption of Cd from soil colloids, facilitating its uptake by roots [63]. Rhizosphere acidification, driven by proton extrusion and the release of low-molecular-weight organic acids such as carboxylates, enhances Cd solubilization and increases its availability for root uptake [64].

In soil solution, Cd is mainly present as a divalent cation (Cd^2+^) [65]. Cadmium enters root tissues through both apoplastic and symplastic pathways. However, transmembrane transport via the symplast is a key regulatory step, as it requires movement through the plasma membrane of root cells. In durum wheat, Cd transport occurs at several cellular interfaces, including the root epidermis and cortex, where nutrient and water uptake are most active, as well as at the vascular transition zones between roots and shoots [66].

Root morphological and anatomical traits significantly affect Cd accumulation. Alterations in root biomass, surface area, and architecture among durum wheat cultivars lead to varying Cd uptake capacities [67]. Studies show that Cd influx is not uniform along the root axis, with higher Cd^2+^ fluxes usually near the root apex, where cells are more metabolically active and membrane transport is intense [68,69]. Some cultivars also display increased Cd^2+^ influx in mature root regions, indicating distinct uptake patterns along the root length [70].

The cation exchange capacity (CEC) of root cell walls further modulates Cd uptake by influencing the binding and mobilization of Cd at the root–soil interface. Enhanced root CEC has been associated with increased Cd mobilization at the plasma membrane, leading to greater Cd entry into root tissues and, ultimately, higher Cd accumulation in aerial organs and grains [71]. Collectively, these findings show that Cd uptake by durum wheat roots is not only determined by external Cd availability but is strongly controlled by rhizosphere chemistry and root structural traits that regulate Cd accessibility and entry into root cells.

### 4.2. Transporter-Mediated Cadmium Uptake in Roots

Because cadmium is not an essential element for plant physiological functions, plants lack Cd-specific uptake systems. Consequently, Cd entry into root cells occurs through transporters and ion channels that normally mediate the uptake of essential mineral nutrients, particularly Fe, Zn, Ca, Cu, and Mn, owing to similarities in ionic charge and radius [72]. In plants, metal uptake is primarily achieved at the root level, whereas intracellular exclusion and detoxification are largely mediated by vacuolar sequestration mechanisms involving specific transporters and chelators. As current knowledge of the genes controlling Cd uptake and homeostasis in durum wheat remains limited. Therefore, evidence from related wheat species, including *T. aestivum* and *T. polonicum*, is discussed here, where appropriate. Future studies should focus on the identification and functional validation of key genes regulating Cd uptake, transport, sequestration, and grain accumulation in this species.

#### 4.2.1. ZIP Family Transporters

Members of the zinc/iron-regulated transporter-like protein (ZIP) family play a major role in Cd uptake at the root plasma membrane. The Arabidopsis iron-regulated transporter 1 (*AtIRT1*), a key component of Fe^2+^ acquisition, also facilitates the uptake of other divalent cations, including Cd^2+^ [73]. In wheat, several ZIP family members have been implicated in Cd influx. For example, the Polish wheat transporters *TpZIP3-2A* and *TpIRT1* are expressed in root tissues and contribute to the uptake of Cd, Fe, and Zn. *TpZIP3-2A* encodes a plasma membrane-localized metal influx transporter whose expression is induced under Fe and Zn deficiency as well as upon exposure to Cd and cobalt [74].

Genome-wide analyses have identified 33 ZIP genes (*TdZIPs*) in wild emmer wheat, many of which are localized to the plasma membrane of root cells and are involved in inorganic ion transport and metabolism [75]. Consistent with these findings, transcriptomic analyses of durum wheat roots exposed to Cd revealed the upregulation of multiple ZIP and zinc-induced facilitator-like (ZIFL) transporter genes, including *ZIFL1*, *ZIF1*, and *ZIP4*, highlighting the central role of ZIP-mediated pathways in Cd entry into root cells [17,76].

#### 4.2.2. NRAMP Transporters

Natural resistance-associated macrophage proteins (NRAMPs) constitute another major transporter family involved in Cd uptake. In plants, NRAMPs are membrane-localized transporters that mediate the uptake and intracellular redistribution of essential metals such as Fe and Mn, andalso facilitate the transport of non-essential and toxic metals, including Cd and Pb [77]. In wheat, 29 *pTaNRAMP* genes have been identified and shown to localize to diverse cellular compartments, including the plasma membrane, mitochondria, chloroplasts, and nucleus of root cells [78].

The plasma membrane-localized transporter *TpNRAMP3* is upregulated in roots and leaves of Polish wheat and has been shown to mediate the uptake of Cd, Co, and Mn in heterologous expression systems. Notably, functional divergence among NRAMP homologs has been observed across species; for instance, *OsNRAMP3* in rice primarily transports Mn, whereas *OsNRAMP1* and *OsNRAMP5* contribute to Cd uptake and root-to-shoot translocation [79]. Similarly, *TpNRAMP5* from Polish wheat mediates Cd, Co, and Mn uptake but not Fe or Zn when expressed in Arabidopsis [80]. In durum wheat, *NRAMP2* expression was strongly induced in the cultivars Svevo and Creso under Cd exposure, supporting its involvement in Cd uptake at the root level [17].

#### 4.2.3. Low-Affinity Cation Transporters

Low-affinity cation transporters (LCTs) also contribute to Cd influx into wheat roots. *TaLCT1*, the first LCT identified in wheat, facilitates the transport of Cd and Ca across the plasma membrane. Heterologous expression of *TaLCT1* in yeast resulted in increased Cd accumulation, confirming its role as a Cd influx transporter [81]. Interestingly, functional variations have been observed among species, as the rice homolog *OsLCT1* primarily acts as a Cd efflux transporter rather than an influx carrier [82].

#### 4.2.4. Calcium-Permeable Nonselective Cation Channels

Cadmium uptake in roots is further mediated by calcium-permeable nonselective cation channels (NSCCs), including hyperpolarization-activated calcium channels (HACC), depolarization-activated calcium channels (DACC), voltage-insensitive cation channels (VICC), and voltage-dependent cation channels (VDCCs) [83]. These channels facilitate the passive influx of monovalent and divalent cations across plant membranes and exhibit moderate permeability to Cd^2+^ [84]. Experimental evidence indicates that Cd^2+^ can enter plant cells through Ca^2+^ channels, particularly in guard cells and root hair apical regions, where Ca^2+^ fluxes are critical for signal transduction and cell growth [85,86].

#### 4.2.5. YSL Transporters and Metal–Chelate Complexes

In addition to free Cd^2+^ ions, Cd may also enter root cells in the form of metal–chelate complexes via Yellow Stripe 1-Like (YSL) transporters. Members of the YSL family mediate the transport of metal complexes bound to phytosiderophores of the mugineic acid family or to the metal chelator nicotianamine (NA) [87]. During phytosiderophore biosynthesis, nicotianamine aminotransferase (NAAT) catalyzes the conversion of nicotianamine to 2′-deoxymugineic acid (DMA), a key chelator involved in metal mobilization [88].

In durum wheat, Cd exposure induces the expression of NAAT and YSL transporters, suggesting an active role for NA- and DMA-mediated pathways in Cd uptake and internal trafficking. Transcriptomic analyses revealed the upregulation of *YSL1* and *YSL2* in Cd-treated durum wheat roots; these transporters are localized to endomembrane systems, including vacuolar membranes, and can transport metal–nicotianamine complexes [17,76].

### 4.3. Long-Distance Cadmium Transport: Xylem Loading and Root-to-Shoot Translocation

Following uptake by root epidermal and cortical cells, Cd is transported to the shoot through the vascular system via tightly regulated long-distance transport processes. The transfer of Cd from roots to aerial tissues depends on its loading into the xylem, translocation driven by transpiration, and subsequent unloading in shoot tissues. In durum wheat, Cd accumulated in root tissues must cross the exodermis and endodermis to reach the stele, where the Casparian strip enforces symplastic transport as a prerequisite for xylem loading [89,90].

Once Cd enters the symplast of root cells, it can be transported to the xylem vessels through transporter-mediated efflux processes. Long-distance Cd transport in wheat is largely driven by transpiration-dependent mass flow, which is proportional to Cd concentration in the soil solution, water flux through the plant, and physiological traits such as root architecture and transpiration rate [91]. Under conditions of elevated Cd availability, apoplastic transport may contribute locally; however, symplastic transport remains the dominant pathway for Cd delivery to the xylem in cereals [92]. Because xylem tracheary elements are dead cells lacking cytoplasmic continuity, Cd must be actively exported from living cells into the xylem apoplast prior to long-distance transport [89].

Key players in xylem loading include members of the P1B-type ATPase family, particularly orthologs of Arabidopsis heavy metal ATPases *AtHMA2* and *AtHMA4*. These transporters facilitate the efflux of divalent metal cations, including Cd^2+^ and Zn^2+^, from the cytoplasm into the xylem by coupling metal transport to ATP hydrolysis [65]. In Arabidopsis, overexpression of *AtHMA4* enhances root-to-shoot translocation of Cd and Zn, whereas disruption of *AtHMA4* activity results in increased metal sensitivity and reduced shoot accumulation [93,94].

In wheat, *TaHMA2b-7A* has been identified as a key regulator of Cd translocation from roots to shoots during the vegetative stage and may also contribute to Cd delivery to developing grains during later growth phases [95]. Furthermore, two alleles of *TdHMA3-B1* are present in various cultivars of durum wheat, with the functional allele being able to transport Cd into the vacuoles and the nonfunctional allele being inactive [96]. The *TdHMA3-B1a*, which is a functional allele, encodes a tonoplast-localized transporter involved in vacuolar sequestration of Cd in root cells. Reduced functionality of *TdHMA3-B1b*, as inferred from heterologous expression studies, limits Cd retention in root vacuoles and consequently promotes enhanced Cd translocation to aerial tissues [97]. These findings highlight the opposing roles of HMA family members in balancing Cd sequestration in roots versus long-distance transport to shoots.

In addition to HMAs, iron-regulated transporters and NRAMP family members contribute to Cd movement beyond the root system. Wheat transporters such as *TpIRT1A* and *TpIRT1B* enhance the translocation of Fe, Mn, Co, and Cd and have been linked to plant growth performance and yield-related traits. These transporters are localized to plasma membranes and intracellular compartments and increase Cd sensitivity when expressed in heterologous systems [98]. Similarly, *TpNRAMP5* and *TtNRAMP6* have been shown to promote Cd accumulation and root-to-shoot transport when overexpressed, with *TtNRAMP6* expression specifically induced by Cd rather than by deficiencies of essential metals [80,99].

Chelation processes further modulate long-distance Cd transport. Complexation of Cd with PCs can enhance Cd mobility within the plant by reducing the cytotoxicity of free Cd^2+^ ions. Cd–PCs complexes are substantially less toxic than free Cd and may facilitate vascular transport while buffering Cd reactivity during translocation [100,101,102]. Moreover, Cd partitioning between roots and shoots is influenced by bidirectional transport processes, including phloem-mediated retranslocation from shoots back to roots, which contributes to whole-plant Cd distribution patterns [103,104,105].

### 4.4. Cadmium Translocation to Grains: Xylem–Phloem Transfer and Node-Based Regulation

**Figure 3:**
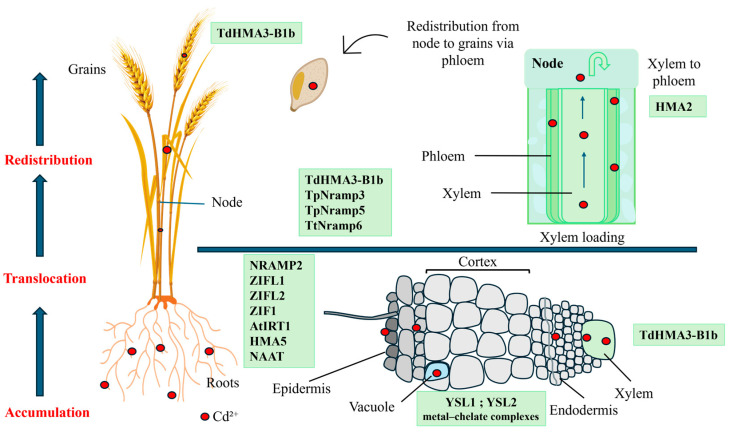
Illustration of the gene’s functions involved in the accumulation, translocation, and redistribution of Cd in durum wheat. Cd accumulation in durum wheat occurs through root absorption, symplasmic movements to xylem, and xylem-to-phloem transportation, and redistribution via phloem in the node. The Cd accumulation by the root is mediated by various genes, including *NRAMP2*, *ZIFL1/2*, *ZIF1*, *AtIRT1*, *HMA5*, and *NAAT*. The translocation of Cd towards the upper parts of the plant is facilitated by *TpNramp3*, *TpNramp5*, and *TtNramp6*. *TdHMA3-B1b* reduced Cd retention in roots and increased Cd transport to shoots and grain.

Cd accumulation in durum wheat grains is the result of synchronized uptake, long-distance transport, and redistribution processes occurring during vegetative and reproductive development (Figure 3). Compared with bread wheat, durum wheat generally accumulates higher Cd concentrations in grains, reflecting differences in root-to-shoot translocation efficiency and internal partitioning mechanisms [106]. Grain Cd loading is influenced by multiple factors, including root uptake capacity, xylem transport efficiency, phloem redistribution, shoot biomass, and root morphological traits [107,108,109].

Root-to-shoot translocation represents a major determinant of grain Cd accumulation in durum wheat. Studies have shown that genotypic variation in Cd content of grains is closely linked to differences in Cd transport efficiency from roots to shoots rather than differences in root uptake alone [110]. During the reproductive phase, Cd accumulated in vegetative tissues can be remobilized and redirected to developing grains. In particular, the early grain-filling stage has been identified as a critical window for Cd translocation, during which leaves and stems contribute substantially to grain Cd content. Wheat leaves have been reported to account for approximately 30% of grain Cd accumulation during early grain filling, although this contribution declines as leaf photosynthetic activity and leaf area decrease at later stages [111].

In contrast to models emphasizing xylem-driven delivery from roots, several studies have demonstrated that phloem-mediated transport plays a dominant role in grain Cd accumulation in durum wheat [112]. The stem node acts as a key regulatory hub for Cd partitioning, facilitating the transfer of Cd from xylem to phloem and thereby directing Cd toward reproductive organs [113,114]. Transporters expressed in nodal tissues, such as heavy metal ATPase 2 (*HMA2*), are implicated in mediating Cd transfer from xylem sap into the phloem stream, ultimately contributing to grain loading [115].

Once loaded into the phloem, Cd is redistributed toward developing grains via mass flow driven by osmotic pressure gradients. In addition to leaves, the wheat spikes themselves contribute to Cd delivery during the late grain-filling stage, further increasing grain Cd content [111]. Differences in Cd redistribution from specific shoot organs, including the rachis, internodes, and flag leaves, have been identified as key factors underlying cultivar-dependent variation in grain Cd accumulation [116]. Although Cd concentrations tend to decrease in internodes following anthesis, increased Cd levels in leaves suggest differential remobilization capacities among shoot tissues. Internodes generally exhibit higher Cd remobilization efficiency than leaves, indicating that stem Cd pools represent a major source of Cd delivered to grains [116,117].

Experimental studies using isogenic and near-isogenic lines (NILs) have further demonstrated that Cd uptake by roots continues during grain filling and contributes directly to grain Cd accumulation, in addition to Cd remobilized from vegetative tissues [118]. Conversely, low-Cd durum wheat cultivars, such as ‘Arcola’, exhibit reduced grain Cd accumulation because of limited root-to-shoot Cd transport during flowering and enhanced shoot-to-root retranslocation during early reproductive stages [119]. These findings collectively highlight the dynamic nature of Cd partitioning during grain development and underscore the central role of xylem–phloem transfer and node-based regulation in determining final grain Cd concentrations in durum wheat.

### 4.5. Genetic Determinants of Cadmium Accumulation in Durum Wheat

Genetic variation plays a central role in controlling Cd accumulation and partitioning in durum wheat, particularly regarding root-to-shoot translocation and grain loading. Numerous studies have demonstrated that differences in grain Cd concentration among durum wheat genotypes are largely attributable to genetically regulated mechanisms governing Cd uptake, sequestration, and long-distance transport rather than to environmental factors alone [103,110].

A development in understanding the genetic basis of grain Cd accumulation in durum wheat was the identification of a single major locus, *Cdu-B1*, located on the long arm of chromosome 5B (5BL). This locus accounts for a substantial proportion of the phenotypic variation in grain Cd concentration, explaining up to 80% of the observed differences among genotypes [120]. Although the gene underlying *Cdu-B1* was initially unknown, subsequent studies demonstrated a strong linkage between *Cdu-B1* and the *HMA3-B1* gene, an ortholog of the rice *OsHMA3* gene encoding a P1B-type ATPase involved in vacuolar sequestration of Cd in root cells [121].

Sequencing of *HMA3-B1* from low- and high-Cd accumulating durum wheat genotypes revealed a 17 bp duplication in high-Cd lines, resulting in a premature stop codon and loss of transporter function. This mutation compromises vacuolar Cd sequestration in roots, thereby enhancing Cd translocation to shoots and grains [121]. These findings strongly support *HMA3-B1* as the contributing gene underlying the *Cdu-B1* locus and establish vacuolar Cd retention in roots as a primary determinant of grain Cd accumulation in durum wheat.

In addition to *Cdu-B1*, minor quantitative trait loci (QTLs) contributing to grain Cd accumulation have been identified. A secondary QTL, designated QCdu.usw-B2, was mapped on chromosome 5BL using an advanced genetic map; however, its contribution to phenotypic variation is relatively small compared with that of *Cdu-B1* [121]. Furthermore, Oladzad-Abbasabadi et al. [122] reported the identification of a novel major QTL for Cd uptake on chromosome arm 5BL, distinct from *Cdu-B1*. This locus was associated with a candidate gene homologous to an aluminum-induced protein (AIP), proposed as *Cdu2*, potentially involved in heavy metal transport and stress responses.

Genome-wide QTL mapping approaches have further expanded the understanding of genetic architecture underlying Cd accumulation in wheat. Cheng et al. [123] identified multiple QTLs associated with Cd uptake, transport, and tissue-specific accumulation in Polish wheat varieties, including a candidate gene, *TpCCX2-4B*, encoding a cation/Ca^2+^ exchanger localized to the plasma membrane and endoplasmic reticulum. Overexpression of *TpCCX2-4B* was associated with reduced Cd uptake and translocation, resulting in lower grain Cd concentrations, particularly in dwarf Polish wheat genotypes.

Molecular marker development has enabled the translation of genetic knowledge into practical breeding tools. Several markers linked to grain Cd concentration, including Cad-5B, Ex_c1343_2570756, and Ex_c17754_26503892, have been shown to reliably predict the low-Cd phenotype in durum wheat populations [124,125]. Among these, Ex_c1343_2570756 has emerged as the most robust marker for selecting low-Cd genotypes and is currently considered highly valuable for marker-assisted selection in durum wheat breeding programs.

These genetic studies demonstrate that grain Cd accumulation in durum wheat is predominantly controlled by a small number of major-effect loci, complemented by additional minor QTLs and modifier genes. The identification of causal genes, such as *HMA3-B1a*, together with tightly linked molecular markers, provides a strong foundation for developing low-Cd durum wheat cultivars through conventional breeding and molecular-assisted approaches (Table 1).

**Table 1:** Transporters and genes encoding the transporters involved in Cd uptake and transport in durum wheat.

Transporter Family	Gene	Expression Site	Subcellular Localization	Function	Reference
ZIP	*AtIRT1*	Root	Vacuole membrane	Central role in Fe^2+^ acquisition and uptake of other divalent cations, including Cd^2+^	[73]
	*ZTP29*	Root	Endoplasmic reticulum	Cd and Zn transport	[17,126]
HMAs	*HMA5*	Root	Plasma membrane	Translocate heavy metals such as Cd and Pb from the symplast into the xylem	[76]
	*TdHMA3-B1b*	Root	-	Reduced Cd retention in roots and increased Cd transport to shoots and grain	[96]
NRAMPs	*NRAMP2*	Root	Intracellular membranes	Metal ion transport	[17]
	*TpNramp3*, *TpNramp5*, and *TtNramp6*	Roots, shoots and grains	Plasma membrane	Cd accumulation in *T. polonicum* and *T. durum*.	[127]
ABC	*TtABCC3a*, *TtABCC3P*	Roots	Tonoplast	Cd Accumulation in grains	[128]
YSL	*YSL1*, *YSL2*	Roots	Vacuole membrane	Regulate Cd entrance and compartmentalization in durum wheat roots	[17]
LCT	*LCT1*	Roots, leaves	Plasma membrane	Facilitates Cd uptake into root cells	[129]

ZIP: zinc/iron-regulated transporter-like protein; HMAs: heavy metal ATPases; NRAMPs: natural resistance-associated macrophage proteins; ABC: ATP-binding cassette transporters; YSL: Yellow-Stripe 1-Like proteins; LCT: low-affinity cation transporter.

## 5. Molecular Responses to Cd Exposure and Detoxification Mechanisms

### 5.1. Transcriptional Regulation of Cadmium Detoxification

Plant responses to Cd exposure involve extensive transcriptional reprogramming aimed at limiting Cd toxicity, maintaining cellular homeostasis, and sustaining growth under stress conditions. Transcription factors (TFs) play a central role in coordinating these responses by regulating genes involved in metal transport, chelation, antioxidant defense, and hormone signaling. Among the TF families implicated in Cd detoxification, WRKY, basic helix–loop–helix (bHLH), and ethylene-responsive factors (ERFs) have emerged as major regulators in both model plants and wheat species [130,131].

#### 5.1.1. WRKY Transcription Factors

WRKY transcription factors are key components of plant signaling networks that regulate stress-responsive gene expression. These TFs can function as transcriptional activators or repressors and are rapidly induced by abiotic stress, including heavy metal exposure [132]. Several WRKY members have been shown to modulate Cd tolerance by controlling genes involved in metal chelation, transport, and antioxidant defense.

In *Arabidopsis thaliana*, *AtWRKY12* indirectly limits Cd accumulation by repressing genes associated with PCs biosynthesis, thereby modulating Cd detoxification capacity [133]. In wheat, *TaWRKY22* directly binds to the promoter of the copper transporter gene *TaCOPT3D*, regulating root responses to Cd stress and contributing to metal homeostasis [134]. Similarly, *TaWRKY74* enhances Cd tolerance by downregulating Cd transporter genes while simultaneously inducing components of the ascorbate–glutathione antioxidant system [135].

Transcriptomic analyses in durum wheat roots exposed to Cd revealed the upregulation of multiple WRKY genes, including *WRKY18*, *WRKY30*, *WRKY33*, *WRKY41*, and *WRKY53*, highlighting their involvement in early Cd-responsive signaling pathways [17]. Functional studies further suggest that WRKY TFs may regulate hydrogen sulfide (H_2_S) signaling, which contributes to Cd tolerance by modulating redox balance and stress signaling cascades [136]. Importantly, the induction of WRKY genes in response to Cd is often transient, with peak expression occurring shortly after exposure, emphasizing the importance of sampling time in transcriptional studies [137].

#### 5.1.2. bHLH Transcription Factors

The basic helix–loop–helix (bHLH) family represents a major group of TFs involved in metal homeostasis and Cd detoxification. Many bHLH TFs are preferentially expressed in root tissues, where they regulate genes associated with metal uptake, chelation, and intracellular sequestration [131].

In Arabidopsis, the coordinated action of *bHLH38*, *bHLH39*, and the FER-like iron deficiency-induced transcription factor (FIT) activates the expression of genes such as *HMA3*, *IRT2*, *MTP3*, and *IREG2*, which collectively contribute to Cd detoxification and metal homeostasis [138]. This regulatory module also induces nicotianamine synthase (NAS) genes, promoting the synthesis of nicotianamine, a key metal chelator that mitigates Cd toxicity by stabilizing metal distribution and sustaining iron homeostasis.

In durum wheat, transcriptomic analyses of NILs with contrasting grain Cd accumulation revealed genotype-specific regulation of bHLH TFs, including *bHLH29* and *bHLH38* [13]. These TFs coordinately regulate genes involved in the biosynthesis of metal chelators, such as NAS, nicotianamine aminotransferase (NAAT), and deoxymugineic acid (DMA), as well as the metal transporter *IREG2*. Additional bHLH-related regulators, including FIT, *bHLH38*/*ORG2*, and *bHLH47*/*PYE*, were also induced by Cd exposure in durum wheat roots, reinforcing the central role of the bHLH–FIT regulatory network in Cd detoxification [17].

#### 5.1.3. Ethylene-Responsive Factors (ERFs)

Ethylene-responsive factors (ERFs) integrate hormonal signaling with abiotic stress responses and play an important role in Cd tolerance. ERFs regulate downstream gene expression either by directly binding to target promoters or through interactions with other TFs, thereby modulating antioxidant defenses, metal transport, and stress-adaptive metabolism [139].

In wheat, Cd exposure induces the expression of tae-miR9670, a Triticeae-specific microRNA whose overexpression enhances Cd tolerance and reduces grain Cd accumulation by activating genes involved in ROS scavenging, detoxification, and metal transport [140]. In Arabidopsis, several ERF genes, including *ERF1*, *ERF2*, and *ERF5*, are transcriptionally activated in response to Cd stress [141].

In durum wheat, ERF-mediated regulation is exemplified by *TdSHN1*, an ERF transcription factor rapidly induced within 24 h of Cd exposure. *TdSHN1* overexpression confers enhanced tolerance to Cd, Cu, and Zn by reducing ROS accumulation and stimulating antioxidant enzyme activities, including superoxide dismutase, peroxidase, and catalase [142]. Promoter analysis of *TdSHN1* revealed multiple heavy metal-responsive cis-elements, supporting its role as a central regulator of metal stress responses. Functional studies further demonstrated that *TdSHN1* modulates genes involved in osmoprotection and stress adaptation, such as *NtP5CS*, which participates in proline biosynthesis [143].

### 5.2. Chelation-Based Detoxification Mechanisms

Chelation is a primary molecular strategy employed by plants to mitigate cadmium toxicity by reducing the concentration of free Cd^2+^ ions in the cytosol and facilitating their safe sequestration within intracellular compartments. In durum wheat, Cd chelation is mainly mediated by sulfur-containing ligands, such as phytochelatins and glutathione, as well as by nitrogen-containing chelators, including nicotianamine (NA) and mugineic acid family phytosiderophores. These chelation-based mechanisms operate in close coordination with transcriptional regulation and transporter-mediated compartmentalization to maintain metal homeostasis under Cd stress.

#### 5.2.1. Phytochelatins and Glutathione-Dependent Detoxification

Phytochelatins are low-molecular-weight, cysteine-rich peptides synthesized enzymatically from glutathione (GSH) by phytochelatin synthase (PCS) in response to Cd exposure. PCs bind Cd^2+^ ions through the thiol groups of cysteine residues, forming Cd–PCs complexes that significantly reduce the reactivity and toxicity of free Cd^2+^ in the cytosol [144]. These complexes are subsequently transported into the vacuole, where Cd is sequestered away from metabolically active cellular processes. Cd exposure triggers rapid activation of GSH biosynthesis and PCs production, reflecting the central role of sulfur metabolism in Cd detoxification. Transcriptomic and biochemical studies have demonstrated that enhanced PCs synthesis is associated with reduced Cd translocation to aerial tissues, thereby limiting Cd accumulation in shoots and grains. The availability of GSH is therefore a critical determinant of Cd tolerance, as it serves both as a direct antioxidant and as a substrate for PCs synthesis.

Among various cultivars of durum wheat, high grain Cd accumulators exhibit lower rates of root and shoot Cd accumulation and root PCs accumulation. However, root-internal PCs chain length distributions and PCs–thiol-to-Cd molar ratios did not significantly differ between cultivars, suggesting that differential grain Cd accumulation is not due to differential PCs-based Cd sequestration in the roots [102]. Further biochemical and molecular studies are crucial for identifying and documenting the role of PCs in plant-internal Cd transport.

#### 5.2.2. Nicotianamine and Mugineic Acid-Mediated Chelation

In addition to sulfur-based chelators, nitrogen-containing ligands play an important role in Cd detoxification and internal metal trafficking in durum wheat. Nicotianamine is a non-proteinogenic amino acid that forms stable complexes with divalent metal ions, including Cd, Fe, and Zn, thereby contributing to metal buffering and redistribution within plant tissues. Cd exposure in durum wheat induces the transcriptional activation of nicotianamine synthase (NAS) genes, including *NAS2*, *NAS3*, and *NAS4*, leading to increased NA production [13].

Nicotianamine serves as a precursor for the biosynthesis of mugineic acid family phytosiderophores, which are synthesized through the coordinated action of nicotianamine aminotransferase (NAAT) and downstream enzymes producing deoxymugineic acid (DMA) [145,146,147]. Although mugineic acids are classically associated with iron acquisition in graminaceous plants, cumulative evidence indicates that NA- and DMA-mediated chelation also contributes to Cd complexation and detoxification, particularly in root tissues. Cd–NA complexes are subsequently trafficked to intracellular compartments, where they can be sequestered via transporter-mediated mechanisms.

#### 5.2.3. Integration with Antioxidant Defense and Sulfur Metabolism

Chelation-based detoxification is tightly linked to the activation of antioxidant defense systems. Cd-induced chelation processes increase the demand for reduced sulfur and redox equivalents, necessitating coordinated regulation of sulfur assimilation and recycling pathways. In durum wheat, Cd exposure enhances the activity of the ascorbate–glutathione (ASC–GSH) cycle in both roots and leaves, even in tissues that accumulate relatively low Cd concentrations [41]. This systemic activation of antioxidant defenses limits Cd-induced oxidative damage and preserves cellular redox balance.

Boubakri et al. [148] identified and characterized the thioredoxin h-type gene family in *T. durum* in response to natural and environmental factor-induced oxidative stress. Furthermore, this thioredoxin h-type TdTrxh2 protein is upregulated in roots and leaves of durum wheat under abiotic stress conditions. Heterologous expression of *TdTrxh2* conferred tolerance against cadmium toxicity stresses by inducing antioxidant enzymes, essentially peroxidase and catalase enzymes [149].

Moreover, Cd stress stimulates transcriptional activation of genes involved in the methionine salvage and sulfur recovery pathways, ensuring sustained production of sulfur-containing metabolites required for GSH and PCs biosynthesis. This metabolic reprogramming highlights the importance of sulfur homeostasis in supporting long-term Cd tolerance and detoxification.

Collectively, chelation-based mechanisms form a central component of Cd detoxification in durum wheat by buffering cytosolic Cd^2+^ levels, facilitating vacuolar sequestration, and reinforcing antioxidant defenses. These processes act synergistically with transcriptional regulation and transporter-mediated compartmentalization to maintain cellular homeostasis under Cd stress.

### 5.3. Transporter-Mediated Cadmium Sequestration and Compartmentalization

**Figure 4:**
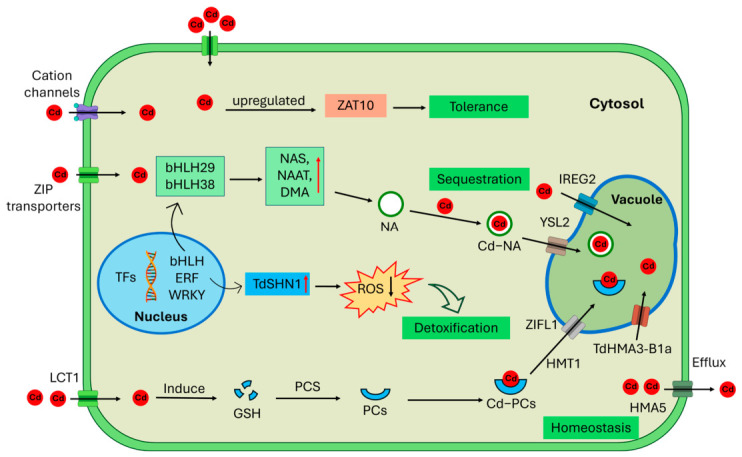
Schematic illustration of Cd detoxification mechanisms in durum wheat cells. Cd enters the cytosol via cation channels and membrane transporters, ZIP, NRAMP, and LCT1. Transcription factors (TFs) such as bHLH, ERF, and WRKY regulate the expression of downstream genes like bHLH29 and bHLH38, which induce the synthesis of metal chelators, including nicotianamine (NA) via NAS, NAAT, and DMA pathways. Cd-NA complexes are formed and sequestered into the vacuole through transporters such as YSL2. The *TdSHN1* gene reduces reactive oxygen species (ROS), thereby contributing to detoxification. Glutathione (GSH) induces the synthesis of phytochelatins (PCs) via PCS, which bind Cd to form Cd-PC complexes, further transported into the vacuole by *HMT1* and *ZIFL1*. Cd efflux is mediated by *HMA5* to maintain homeostasis. *ZAT10* is upregulated, enhancing plant tolerance. Collectively, these mechanisms contribute to Cd detoxification, sequestration, and cellular homeostasis.

Following chelation in the cytosol, effective detoxification of Cd in durum wheat requires its compartmentalization into intracellular organelles, primarily the vacuole. This process is mediated by specialized membrane transporters that facilitate the sequestration of Cd as free ions or chelates, thereby preventing interference with essential metabolic processes and limiting cellular Cd toxicity, as illustrated in Figure 4. Unlike the transport processes described in Section 4, which regulate Cd movement between plant organs and its allocation to aerial tissues and grains, the mechanisms discussed here primarily operate at the cellular level and aim to detoxify Cd through intracellular sequestration, compartmentalization, and maintenance of metal homeostasis.

In durum wheat, Cd resistance is closely linked with the regulation of genes encoding metal transporters involved in intracellular sequestration and detoxification (Table 2). Key transporter genes, including *TdNRAMP*, *TdHMT1*, *TdHMA5*, *TdZIF1*, *TdZIFL2*, and *TdZTP29*, contribute to Cd tolerance by facilitating the transport and compartmentalization of Cd within root cells. Enhanced expression of these transporters promotes Cd sequestration into root vacuoles, thereby immobilizing the metal in root tissues and limiting its translocation to aerial organs [150].

#### 5.3.1. ABC Transporters and MRP-Mediated Vacuolar Sequestration

ATP-binding cassette (ABC) transporters, particularly members of the ABCC (multidrug resistance-associated protein, MRP) subfamily, represent a major detoxification route for Cd in plants. Microarray analyses of near-isogenic durum wheat lines differing in grain Cd accumulation identified transcripts encoding putative MRP/ABCC transporters. Subsequent molecular characterization associated these transcripts with *TtABCC3*, a tonoplast-localized ABCC transporter involved in vacuolar sequestration of Cd complexes and the regulation of Cd accumulation in durum wheat [128]. Consistent with these findings, *TaABCC1* has been implicated in vacuolar sequestration of Cd in wheat, thereby contributing to cellular detoxification and limiting Cd accumulation in aerial tissues [151]. Functional homologs in *A. thaliana*, such as *AtMRP1* and *AtMRP2*, mediate the vacuolar import of phytochelatin–Cd complexes, underscoring the evolutionary conservation of ABCC-mediated Cd detoxification mechanisms [152]. In addition, ABC transporters belonging to the ABCG (PDR) subfamily, such as *AtPDR8*, contribute to Cd resistance by exporting Cd or Cd conjugates across the plasma membrane, thereby reducing intracellular Cd burden [153].

#### 5.3.2. HMT1 and Phytochelatin-Dependent Transport

The heavy metal tolerance factor (HMT1) is an ATP-dependent transporter involved in the vacuolar sequestration of Cd–phytochelatin (Cd–PC) complexes. In durum wheat, homologs of HMT1 are preferentially upregulated in low-Cd-accumulating genotypes, supporting their role in restricting cytosolic Cd availability and enhancing cellular tolerance [13]. Increased expression of HMT1 is frequently accompanied by induction of genes encoding peroxidases, suggesting coordinated activation of vacuolar sequestration, antioxidant defenses, and cell wall strengthening as adaptive responses to Cd stress.

By mediating the transport of Cd–PC complexes into the vacuole, HMT1 provides a crucial link between chelation-based detoxification and transporter-mediated compartmentalization, thereby preventing Cd interference with cytosolic enzymes and metabolic pathways.

#### 5.3.3. HMA and IREG Transporters in Vacuolar Cd Storage

Tonoplast-localized P1B-type ATPases also play a central role in Cd detoxification by mediating metal sequestration into vacuoles. In durum wheat, *TdHMA3-B1a* contributes to phenotypic variation in Cd tolerance by facilitating the transport of Cd and Zn into root vacuoles, thereby reducing cytosolic Cd toxicity [96]. While *HMA3* has been discussed in Section 4.5 as a major genetic determinant of grain Cd accumulation, its role in the present context is considered at the cellular level, where *HMA3* primarily functions in vacuolar Cd sequestration in root cells, contributing to detoxification and tolerance.

In addition to HMAs, the iron-regulated transporter *IREG2*, originally characterized for its role in iron homeostasis, is strongly induced under Cd stress in durum wheat roots, particularly in low-Cd genotypes [13,96]. The elevated expression of *IREG2* suggests its involvement in vacuolar Cd storage and highlights the functional overlap between iron and Cd detoxification pathways.

#### 5.3.4. ZIFL and YSL Transporters in Cd–Chelate Trafficking

Zinc-induced facilitator-like (ZIFL) transporters further contribute to Cd detoxification by mediating the transport of divalent cations and metal–chelate complexes across the tonoplast. In durum wheat, *ZIFL1* and *ZIFL2*, which share high sequence similarity with *ZIF1*, are highly expressed in low-Cd genotypes and are associated with enhanced vacuolar compartmentalization of Cd^2+^ [76]. These transporters likely function alongside metal chelators to promote the stabilization of Cd within vacuoles.

Members of the Yellow Stripe 1-Like (YSL) transporter family, particularly *YSL2*, also play a key role in Cd detoxification. In low-Cd durum wheat genotypes, such as Creso and low-Cd NILs, increased expression of *YSL2* is associated with reduced Cd accumulation in leaves and grains [76,154]. *YSL2* is localized to endomembrane systems, including the vacuolar membrane, and mediates the transport of Cd–nicotianamine complexes, thereby linking chelation-based detoxification to transporter-mediated sequestration.

**Table 2:** Genes involved in Cd detoxification and/or homeostasis in durum wheat.

Transporter Family	Gene	Expression Site	Subcellular Localization	Function	Reference
ZIP	*ZIP4*	Root	Plasma membrane	Zn/Cd uptake and homeostasis	[17]
ZIFL	*ZIFL1* and *TdZIFL2*	Root	Vacuole membranes	Compartmentalization of Cd into vacuoles	[76]
HMAs	*TdHMA3-B1a*, *TdHMA5*	Roots, grains	Tonoplast	Transport Cd and Zn into vacuoles, reducing Cd accumulation in shoots and grains	[96,154]
ABC	*TtABCC3a*, *TtABCC3P*	Root	Tonoplast	Regulate Cd uptake or transport within the plant	[128]
	*HMT-1*	Root	Tonoplast	Sequesters Cd–PC complexes into vacuoles, thereby reducing cadmium toxicity	[13]
YSL	*YSL1*, *YSL2*	Root	Vacuole membrane	Regulate Cd entrance and compartmentalization in durum wheat roots	[17]
MRP	-	Root	Tonoplast	Sequester Cd in the vacuole	[128]
LCT	*LCT1*	Roots, leaves	Plasma membrane	Facilitates Cd uptake into root cells	[129]
	*IREG2*	Root	Tonoplast	Cd compartmentalization in the vacuole	[13]
	*NAS2*, *NAS3*, and *NAS4*	Root	-	Synthesize NA, a metal chelator that binds Cd to facilitate vacuolar sequestration	[76]
	*TdTrxh2*	Roots and leaves	Cytoplasm	TdTrxh2 promotes Cd tolerance through the redox homeostasis regulation and its protective role.	[148,149]
	*TdHMT1*, *TdZIF1*, *TdZTP29*	Root	Tonoplast	Promotes Cd sequestration in root vacuoles	[154]
NRAMP	*TdNRAMP*				

ZIP: zinc/iron-regulated transporter-like protein; ZIFL: zinc-induced facilitator-like; HMAs: heavy metal ATPases; NRAMPs: natural resistance-associated macrophage proteins; ABC: ATP-binding cassette transporters; YSL: Yellow-Stripe 1-Like proteins; LCT: low-affinity cation transporter.

## 6. Conclusions and Future Perspectives

Cadmium accumulation in durum wheat is governed by a complex network of transporters, chelators, and regulatory factors that control Cd uptake, root-to-shoot translocation, intracellular sequestration, and grain loading. Among these, HMA, NRAMP, ZIP, YSL, and ABCC transporters, together with PCs, NA, and transcriptional regulators, play central roles in modulating Cd homeostasis and mitigating Cd toxicity. Recent transcriptomic and genetic studies have identified key candidate genes associated with low grain Cd accumulation, providing valuable targets for breeding programs aimed at improving food safety.

Despite these advances, several molecular mechanisms remain insufficiently characterized in durum wheat, and many regulatory pathways have been inferred from studies in related wheat species. Future research should prioritize the functional validation of candidate genes, the elucidation of species-specific regulatory networks, and the integration of genomics, transcriptomics, and gene-editing approaches to accelerate the development of low-Cd durum wheat cultivars. Such efforts will contribute to sustainable wheat production and reduce Cd exposure through the food chain.

## Data Availability

No new data were created or analyzed in this study. Data sharing is not applicable to this article.

## Abbreviations

The following abbreviations are used in this manuscript:

PCs	Phytochelatins
ROS	Reactive oxygen species
CEC	Cation exchange capacity
ZIP	Zinc/iron-regulated transporter-like protein
NRAMPs	Natural resistance-associated macrophage proteins
LCTs	Low-affinity cation transporters
NA	Nicotianamine
QTLs	Quantitative trait loci
ABC	ATP-binding cassette transporters
bHLH	Basic helix–loop–helix
